# Effects of the dopamine depleting agent tetrabenazine in tests evaluating different components of depressive-like behavior in mice: sex-dependent response to antidepressant drugs with SERT and DAT blocker profiles

**DOI:** 10.1007/s00213-023-06412-9

**Published:** 2023-07-05

**Authors:** Carla Carratalá-Ros, Andrea Martínez-Verdú, Regulo Olivares-García, John D. Salamone, Mercè Correa

**Affiliations:** 1 Àrea de Psicobiologia, Universitat Jaume I, Campus de Riu Sec, 12071 Castelló, Spain; 2 Behavioral Neuroscience Div, University of Connecticut, Storrs, CT 06269-1020 USA

**Keywords:** Sex differences, Bupropion, Fluoxetine, Anergia, Fatigue, Depression, Rodent models

## Abstract

**Background:**

Depression is a disorder twice as common in women than in men. There are sex differences in the symptomatology and treatment response to this disorder. Impairments in behavioral activation (i.e. anergia, fatigue) are often seen in people with depression and are highly resistant to treatment. The role of mesolimbic dopamine (DA) in regulating behavioral activation has been extensively studied in male rodents, but little is known in female rodents.

**Objective:**

The present studies assessed potential sex differences in rodent paradigms used to study different components of depressive-like behavior, and in the treatment response to antidepressants with different mechanisms of action.

**Methods:**

Male and female CD1 mice received Tetrabenazine (TBZ), a VMAT-2 blocker that depletes DA and induces depressive symptoms in humans. Mice were tested on the Forced Swim Test, (FST), the Dark–Light box (DL), the elevated plus maze (EPM), Social Interaction (SI) test, and sucrose preference and consumption using the two bottles test. In addition, bupropion (a DA reuptake inhibitor) or fluoxetine (a serotonin reuptake inhibitor) were used to reverse TBZ-induced anergia.

**Results:**

In the FST, bupropion reversed TBZ effects in both sexes but fluoxetine was only effective in female mice. DA depletion did not affect other aspects of depression such as anxiety, sociability or sucrose consumption, and there was no interaction with bupropion on these parameters. In TBZ treated-females SERT-blockers may be effective at reversing anergia in aversive contexts (FST), and potentiating avoidance of anxiogenic stimuli.

**Conclusions:**

Pro-dopaminergic antidepressants seem more efficacious at improving anergia in both sexes than SERT-blockers.

## Introduction

Preclinical studies using animal models involve behavioral tests that are thought to reflect common symptoms seen in psychiatric disorders. These studies typically use male rodents, and usually avoid performing the same experiments in female rodents (Dalla et al. 2010; Zucker and Beery 2010). This is also true for the study of depression, despite the fact that depression is twice as frequent in women compared to men (Silverstein 2002; Kessler 2003; Parker and Brotchie 2010; Seedat et al. 2010). Moreover, there are sex differences in the progress and the symptomatology of this disorder (Thiels et al. 2005; Marcus et al. 2005; Gorman 2006). Some of the most common symptoms of depression are energy-related dysfunctions such as slowness and self-reported fatigue (Tylee 1999; Salamone and Correa 2012). Fatigue seems to be a symptom that establishes clear sex differences among depressed patients. It has been observed that some symptoms categorized as "somatic", such as fatigue, are more common among women (Dekker et al. 2007; Bjornelv et al. 2011), and this difference increases with age (Silverstein 2002). In addition, women diagnosed with depression usually express greater lack of activity than men (Breslin et al. 2006; Poutanen et al. 2009). Moreover, clinical studies have found differences between women and men in terms of the response to antidepressants. Thus, it seems that antidepressants that inhibit the serotonin transporter (SERT) such as fluoxetine (FLX) or citalopram have a better outcome in women, while men are more likely to be more responsive to tricyclic antidepressants (Kornstein et al. 2000; Khan et al. 2005; Young et al. 2009).

One of the animal behavioral models that has been used to study differences between male and female rodents is the Forced Swim Test (FST). This paradigm evaluates the attempt of rodents to escape in a vigorous way from a stressful non-escapable situation, being in this case a deep tank filled with water (Porsolt et al. 1977). Eventually, animals will cease the vigorous attempts to escape and will passively float in the tank (Porsolt et al. 1977). Thus, immobility is the classical parameter that is measured, and antidepressants have repeatedly been shown to reverse this immobility (Armario et al. 1988; Petit-Demouliere et al. 2005; Costa et al. 2013). Studying sex differences in the FST, a few studies have found that female rodents have higher immobility time in comparison with male rodents (Kokras et al. 2012, 2015), and this effect was alleviated in female rodents following the administration of SERT inhibitors. In terms of their immobility response, female rats and mice are more responsive to SERT inhibitors than male rodents (Jones and Lucki 2005; Dalla et al. 2010; Kokras et al. 2015; Fernández-Guasti 2017). Other behaviors that can be evaluated in the FST are swimming and climbing. Both behaviors are considered active behaviors that also are modified by antidepressants (Armario et al. 1988; Lucki 1997), and are likely to be affected by DA manipulations more than the traditional immobility measure (Gil and Armario 1998; Costa et al. 2013). However, little is known about the differences between male and female rodents in the execution of these two behaviors. Some animal studies have found that, under basal conditions, female rodents display less climbing than male rodents, suggesting that females are less active than males, and antidepressant drugs were able to enhance climbing behavior in female rodents (Martínez-Mota et al. 2011; Kokras et al. 2015). Other studies failed to detect sex differences on climbing and swimming in the FST (Verma et al. 2010; Mourlon et al. 2010). Moreover, to our knowledge, there are no studies that have studied the effects of dopaminergic drugs on modulation of climbing in female rodents assessed in the FST.

A pharmacological manipulation that is used to model features of depression is the drug tetrabenazine (TBZ). TBZ acts to deplete monoamines by inhibiting the vesicular monoamine transporter-type 2 (VMAT-2), and at low doses this drug has its greatest effects on ventrostriatal DA (Pettibone et al. 1984; Nunes et al. 2013b; López-Cruz et al. 2018). In male rats, TBZ reduced DA levels in Nucleus accumbens around 60% (Nunes et al. 2013b). In that study, when rats were tested on procedures involving effort-related decision making, TBZ reduced selection of high-effort activity and increased selection of more sedentary options. Thus, TBZ produces a decrease in effortful activities such as lever pressing at high ratios in operant tasks to get palatable reinforcers, but increases the consumption of free available chow or free diluted sucrose (Nunes et al. 2013b; Pardo et al. 2015; Rotolo et al. 2019). Similarly, in previous studies from our laboratory in male mice, we have demonstrated that the effective dose of TBZ (8 mg/kg) on effort-based decision-making studies, reduced ventral striatal DA levels around 65% (López-Cruz et al. 2018). Moreover, TBZ also partially shifts behavior in a choice T-maze task in male mice, reducing vigorous activities such as time running in a running wheel but increasing time eating sucrose pellets (López-Cruz et al. 2018; Carratalá-Ros et al. 2020; 2021a, b). In the FST, TBZ reduces climbing in male mice, and increased immobility time (Carratalá-Ros et al. 2020, 2021a, b). Moreover, these effects in males were reversed by antidepressants that block the DA transporter (DAT) (Randall et al. 2015), but not by serotonin uptake (SERT) inhibitors (Carratalá-Ros et al. 2021a, b).

Thus, the aim of the present study is to explore the ability of two antidepressants with different mechanisms of action; bupropion (catecholamine uptake blocker, with greater actions on extracellular DA and norepinephrine), and fluoxetine (SERT inhibitor) to reverse the effect produced by TBZ in male and female mice tested in paradigms that evaluate exertion of effort to escape a stressful situation such as the FST (Carratalá-Ros et al. 2020, 2021a, b). Anxiety, social avoidance and lack of enjoyment are symptoms that can also be seen, at different levels and with different incidence, in depressed patients (Nutt 1999; Dekker et al. 2007; Poutanen et al. 2009; Cuthbert and Insel 2013). Thus, we additionally assessed the impact of these two antidepressants on the effects of TBZ using paradigms that evaluate behaviors shaped by emotional factors, such as responses in anxiogenic environments (dark and light box; DL, and elevated plus maze; EPM), or avoidance of novel social stimuli (evaluated in a social interaction task). Finally, we evaluated preference and consumption of liquid sucrose, which is often related to anhedonia-like behaviors, but can also be interpreted as avoidance of salient palatable stimuli associated with sickness. Based on previous studies in males (Carratalá-Ros et al. 2020, 2021a, b) we expect that TBZ will not affect measures of anxiety, social preference or sucrose consumption, bupropion will be more efficacious than fluoxetine to reverse the anergic effects of TBZ, and there would be sex differences in the response to these antidepressants.

## Materials and Methods

### Animals

CD1 adult male and female mice (N = 311) purchased from Janvier, France S.A. were 7–9 weeks old (25–50 g) at the beginning of the study. Mice were housed in groups of three or four per cage, with standard laboratory rodent chow and tap water available ad libitum. The colony was kept at a temperature of 22 ^+^ 2 ºC with lights on from 08:00 to 20:00 h. All animals were under a protocol approved by the Institutional Animal Care and Use committee of Universitat Jaume I. All experimental procedures complied with directive 2010/63/EU of the European Parliament and of the Council, and with the “Guidelines for the Care and Use of Mammals in Neuroscience and Behavioral Research”, National Research Council 2003, USA. All efforts were made to minimize animal suffering, and to reduce the number of animals used.

### Pharmacological agents

Tetrabenazine (TBZ; CIMYT Quimica SL, Spain) was dissolved in a vehicle solution of 0.9% saline (80%) plus dimethylsulfoxide (DMSO 20%, final pH 5.5), and administered 120 min before testing. Bupropion hydrochloride (BUP; Alfa Aesar, Spain) and fluoxetine (CIMYT Quimica SL, Spain) were dissolved in 0.9% saline, and administered 30 min before the test started. DMSO (20% v/v) and saline were used as the VEH control treatments. All the substances were administered intraperitoneally (IP). Doses of the three substances and times elapsed after injection of drugs were selected based on previous behavioral work (Carratalá-Ros et al. 2020, 2021a, b) and neurochemical studies (López-Cruz et al. 2018) demonstrating that, in mice, these conditions are optimal for depleting DA and producing behavioral effects.

### Testing procedures

All behavioral procedures began two hours after the light period started. The behavioral test room was illuminated with a soft light, and external noise was attenuated.

#### Forced swim test (FST)

This paradigm is considered to be a model of behavioral despair and is used as a test for assessing depressive-like states and for evaluating drugs with potential as antidepressants (Porsolt et al. 1977). Classically, it involves evaluation of immobility defined when the animal remains motionless, making only minor movements to balance the body and keep the head above the water. In addition, we also assessed escape-related mobility such as climbing or struggling (Armario et al. 1988). Climbing is defined as any energetic vertical movement of all four limbs against the wall of the tank. Mild swimming was recorded when animals carried out horizontal movements with their forepaws, leading to the displacement of the body throughout the swim chamber (Armario et al. 1988). Naïve male and female mice were placed in a transparent cylindrical glass tank (26 cm high and 18 cm diameter) filled with water (14 cm) and maintained at a temperature of 25ºC. Water was changed between animals. During the 6 min test, mice were videotaped from the side, and climbing, immobility and swimming were later measured by an observer unaware of the experimental condition. After the test, mice were dried with a soft towel, put back in a box with absorbant paper under a warming light, and were monitored for 10 min.

#### Dark and light box (DL)

The DL test is based on the conflict between the innate tendency to explore a novel environment and to avoid a brightly lighted open area (Blumstein and Crawley 1983). The DL apparatus consisted of a polypropylene chamber divided in two compartments by a partition containing a small opening (5 cm H × 5 cm W). The light compartment (25 cm W × 25 cm H × 25 cm L) was open, painted in white and illuminated (335 lx), while the dark compartment (25 cm W × 25 cm H × 18 cm L) was painted in black and had a removable ceiling to close it (Kulesskaya and Voikar 2014). To start the test session, mice were individually placed in the dark chamber facing one corner. Test sessions were videotaped, and the total number of crosses and the total time spent in the lit chamber were recorded for 5 min (López-Cruz et al. 2014; Carratalá-Ros et al. 2020).

#### Elevated plus maze (EPM)

The EPM consists of two open and two enclosed arms (65 cm L × 5 cm W) arranged in a plus configuration and intersecting in a central platform. It is made of black polypropylene and is elevated 50 cm above the floor. The open arms have a 1 cm border around their perimeter and the closed arms have a 20 cm translucent wall. This anxiety paradigm measures the avoidance that rodents show to high open spaces. Under normal conditions mice spend more time in, and make more entries into, the closed arms of the maze (Lister 1987). Animals were placed in the central platform with their head pointing at one enclosed arm, and they were assessed during 5 min. Sessions were videotaped and a trained observer registered total time spent in the open arms, and total entries in the 4 arms as an index of locomotion. An entry into an arm was recorded when the animal crossed with all four legs the line that connected that arm with the central platform (procedure is based on López-Cruz et al. 2014).

#### Social interaction and preference test

Social interaction and preference were measured in a three-chambered social testing box (Landauer and Balster 1982). This test is based on the preference of rodents for spending time with a conspecific animal rather than remaining alone or exploring non-social stimuli (File and Hyde 1978; Berton et al. 2006). Every mouse was placed in the center of the chamber of the social interaction apparatus and they freely explored the social arena during 10 min in the presence of a caged conspecific on one side of the compartment, while on the other side there was a small wire cage containing an object. The center compartment was empty. The placement of the conspecific or the object was counterbalanced between animals. A trained experimenter who was unaware of the experimental conditions, manually recorded time spent sniffing each target (conspecific vs object) as a measure of social preference. Crosses between compartments were also registered. Procedures were based on López-Cruz et al. (2016). The social preference index was calculated as the time interacting with the conspecific divided by the total time of interaction (time with the conspecific plus time with the object).

#### Sucrose consumption and preference

The sucrose preference test for rodents (Muscat and Willner 1989; Monleon et al. 1995), based on a two-bottle choice paradigm, is a widely used behavioral paradigm for the evaluation of depressive states in rodents. A decreased intake of, or preference for, sweet solutions is considered to reflect an anhedonic-like behavior (Fonseca-Rodrigues et al. 2022). In an adaptation of this test (see Correa et al. 2020), during 3 days previous to the drug test day, in 60 min sessions, non-food/water restricted mice were individually placed in standard home cages where they had free access to two different liquid sucrose concentrations (10% and 5%) placed in graduated bottles. On the drug test day, the amount of liquid left (ml) of both solutions was registered at the end of the session. Volume consumed of both solutions and sucrose preference index, calculated as the 10% sucrose consumed divided by the total liquid consumed (5% plus 10% sucrose solutions), were evaluated.

#### Statistical Analysis

Normally distributed and homogenous data (according to Kolmogorov–Smirnov test) for the FST, DL, EPM, social interaction, and sucrose liquid intake were evaluated by one-way ANOVA for the factor treatment in each sex and when the overall ANOVA was significant, non-orthogonal planned comparisons using the overall error term were used (Keppel 1991). Additional analyses comparing both sexes with a two-way factorial ANOVA sex x treatment were performed for all the paradigms and dependent variables, in order to determine differences in baseline behavior based on sex. However, only the significant results of this interaction were reported. For these comparisons, α level was kept at 0.05 because the number of comparisons was restricted to the number of treatments minus one. All data were expressed as mean ± SEM, and significance was set at p < 0.05. STATISTICA 7 software was used.

## Results

### Experiment 1. Ability of bupropion and fluoxetine to reverse the effects produced by TBZ on behavioral activation in male and female mice evaluated in the FST

Naïve male (n = 40) and female (n = 40) mice received either vehicle or a combination of TBZ plus bupropion or TBZ plus fluoxetine and were placed in the FST during 6 min after the corresponding lead time had passed. Thus, treatments were: VEH/VEH, 8.0 mg/kg TBZ/VEH, 8.0 mg/kg TBZ/10.0 mg/kg BUP, or 8.0 mg/kg TBZ/20.0 mg/kg FLX.

**Fig. 1:**
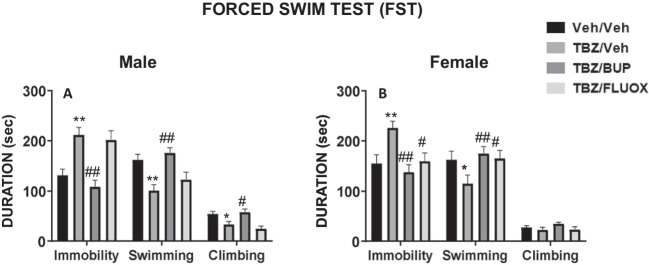
Effect of treatment in male (**A**), and female (**B**) mice on measures of behavioral activation as duration of immobility, swimming, and climbing in the FST assessed during 6 min. Bars represent the mean ± S.E.M of accumulated seconds. *p < 0.05, **p < 0.01 significantly different from Veh/Veh. #*p* < 0.05, ##*p* < 0.01 significantly different from TBZ/Veh

In males (Fig. 1A), the one-way ANOVA for the drug treatment factor showed significant effects on the three dependent variables: immobility time (F(3,36) = 11.37, p < 0.01), time spent swimming (F(3,36) = 7.98, p < 0.01), and time spent climbing (F(3,36) = 7.61, p < 0.01). Planned comparisons revealed that the group that received TBZ/VEH displayed significantly less climbing (p < 0.05), decreased time spent swimming (p < 0.01), and significantly increased immobility time (p < 0.01) compared to the VEH/VEH group. Bupropion reversed the effects of TBZ, decreasing time of immobility (p < 0.01), and increasing time swimming (p < 0.01), and time climbing (p < 0.05), in comparison with the group that received TBZ/VEH. However, co-administration of fluoxetine with TBZ failed to reverse the effects produced by TBZ in males.

In females (Fig. 1B), the one-way ANOVA for the treatment factor showed significant effects on immobility time (F(3,36) = 5.93, *p* < 0.01), and time spent swimming (F(3,36) = 5.65, *p* < 0.01), but no significant effect on time spent climbing (F(3,36) = 1.08, *p* = 0.37). Planned comparisons revealed that female mice that received TBZ/VEH showed significantly increased time spent immobile (*p* < 0.01), and significantly less time swimming (*p* < 0.05) compared to the VEH/VEH group. Bupropion was able to reverse the effects produced by TBZ; thus the TBZ/BUP-treated group displayed significantly less immobility time (*p* < 0.01), and more time swimming (*p* < 0.01) compared to the TBZ/VEH-treated group. In contrast with males, fluoxetine in females was also able to reverse the effects produced by TBZ. Thus, the group that received TBZ/FLX showed significantly increased time spent swimming (*p* < 0.05), and decreased immobility time (*p* < 0.05) in comparison to TBZ/VEH treated female mice.

### Experiment 2. Effect of bupropion or fluoxetine in TBZ-treated male and female mice on anxiety parameters as measured in the DL and EPM paradigms

Four groups of naïve male mice (N = 40) and four groups of naïve female mice (N = 40) received the same combination of treatments (VEH/VEH, 8.0 mg/kg TBZ/VEH, 8.0 mg/kg TBZ/10.0 mg/kg BUP, or 8.0 mg/kg TBZ/20.0 mg/kg FLX) and after the lead time had elapsed, they were placed first in the DL paradigm for 5 min, and immediately after in the EPM paradigm for 5 more minutes.

**Fig. 2:**
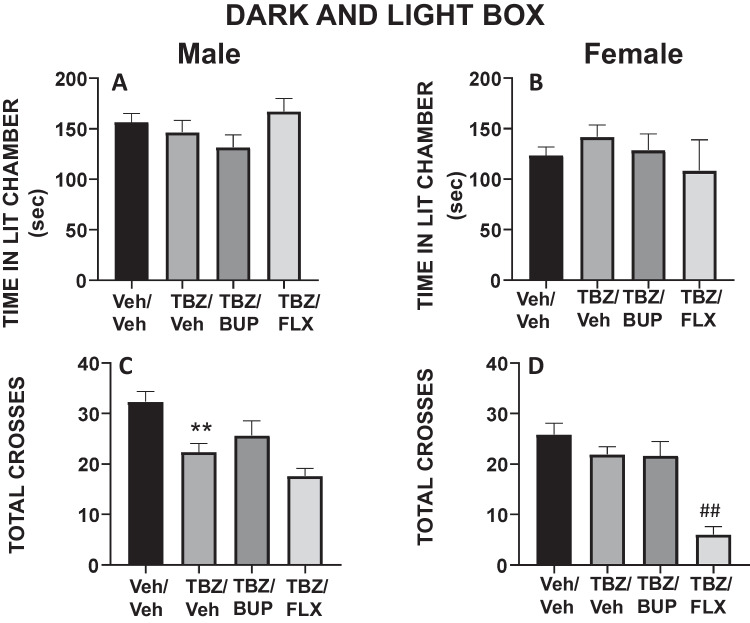
Effect of treatment in male and female mice evaluated in the DL. Time spent in the lit chamber (**A**, **B**), and total of entries between the two compartments (**C**, **D**) of the DL box during 5 min. Bars represent the mean ± S.E.M. of accumulated seconds or number of crosses. **p < 0.01 significantly different from Veh/Veh. ##*p* < 0.01 significantly different from TBZ/Veh

In the DL paradigm experiment, the one-way ANOVA showed that there were no significant effects of treatment on anxiety parameters (assessed as time spent in the illuminated arena) in either male mice (F(3,37) = 1.69, p = 0.18) or female mice (F(3,37) = 0.56, p = 0.64) (Fig. 2A-B). However, one-way ANOVA for the number of crosses between compartments in the DL paradigm revealed significant effects on both; in male mice (F(3,37) = 8.59, p < 0.01), and in female mice (F(3,37) = 16.90, p < 0.01). Planned comparisons indicated that the groups of male mice that received TBZ/VEH displayed significantly fewer crosses than the VEH/VEH group (p < 0.01; Fig. 2C). Among female mice, the combination of TBZ/FLX further impaired the total number of crosses between compartments in comparison to the TBZ/VEH group (p < 0.01; Fig. 2D).

**Fig. 3:**
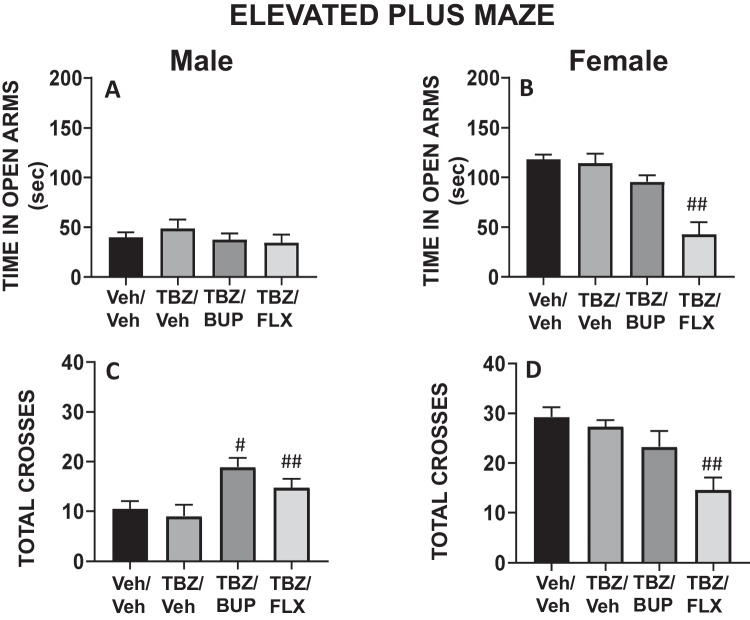
Effect of treatment in male and female mice evaluated in the EPM paradigms. Time spent in the open arms (**A**, **B**), and total of entries to the four arms (**C**, **D**) during 5 min. Bars represent the mean ± S.E.M. of accumulated seconds or number of crosses. *p < 0.05, **p < 0.01 significantly different from Veh/Veh. #*p* < 0.05, ##*p* < 0.01 significantly different from TBZ/Veh

For the EPM tests, in male mice the one-way ANOVA did not show a significant effect of treatment on anxiety parameters evaluated as the time spent in the open arms of the EPM (F(3,37 = 0.70, p = 0.55, Fig. 3A). However, in female mice, the one-way ANOVA did show a significant effect in time spent in the open arms of the EPM (F(3,37 = 14.12, p < 0.01). Planned comparisons revealed that female mice receiving the combination of TBZ/FLX spent significantly less time in the open arms compared to the group that received TBZ/VEH group (p < 0.01; Fig. 3B). In addition, the one-way ANOVA for locomotion in the EPM led to significant results in both sexes (male mice (F(3,37 = 5.52, p < 0.05), female mice (F(3,37 = 7.53, p < 0.01)). Planned comparisons indicated that although TBZ on its own did not produce a significant effect compared to VEH/VEH, male mice receiving the combination of TBZ/BUP or TBZ/FLX significantly increased the total of entries in comparison with the TBZ/VEH group (p < 0.01 for both; Fig. 3C). In the female mice however, the combined TBZ/FLX treatment reduced entries in comparison to the TBZ/VEH group (p < 0.01; Fig. 3D).

In the additional test to account for sex differences, the two-way (sex x treatment) factorial ANOVA for the dependent variable time spent in the open arms lead to a significant interaction (F(3,61) = 6.78, p = 0.0005), and planned comparisons revealed that females who received veh/veh, TBZ/veh and TBZ/BUP spent more time in the open arms than males that received the same drug treatment (p < 0.01 for each comparison). Similar results on the number of crosses were observed. The two-way factorial ANOVA showed an interaction sex x treatment in total crosses to all arms of the EPM (F(3,61) = 10.48, p = 0.00001). Planned comparisons revealed that female mice who received veh/veh and TBZ/veh crossed more in comparison to males that received the same drug treatment (p < 0.01 for all conditions). These data suggest that, at least in this paradigm, males have a higher baseline of anxiety as seen by avoidance to spend time in the open arms.

### Experiment 3. Effect of bupropion or fluoxetine in TBZ-treated male and female mice on social exploration assessed in a three-chamber social preference test

New groups of males (N = 30) and females (N = 28) mice received the same treatment combinations as in previous studies, and they were placed in a social preference box for 10 min.

**Fig. 4:**
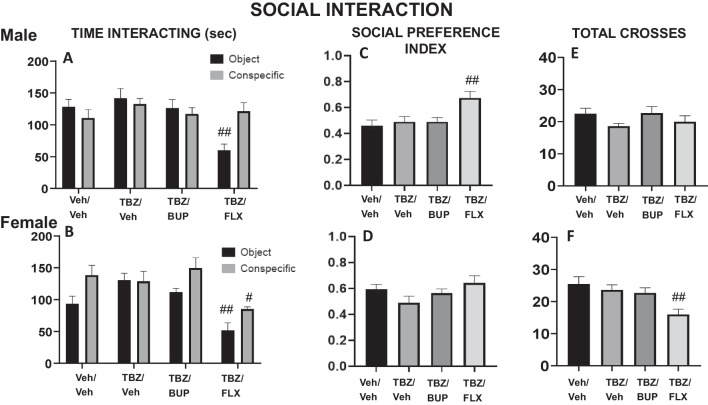
Effect of treatment in male and female mice on social interaction. Time sniffing the conspecific and time sniffing the object (**A**, **B**), social preference index (**C**, **D**), and crosses between the 3 compartments (**E**, **F**) assessed during 10 min. Bars represent the mean ± S.E.M of accumulated seconds or number of crosses. *p < 0.05, **p < 0.01 significantly different from Veh/Veh. ##p < 0.01 significantly different from TBZ/Veh

In males, the one-way ANOVA showed a significant effect of treatment on time spent interacting with the object (F(3,26) = 6.13, p < 0.01), but no significant effect on time spent interacting with the conspecific (F(3,26 = 0.69, p = 0.56). Planned comparisons revealed that male mice that received the combination of TBZ/FLX significantly decreased the time spent interacting with the object in comparison with the TBZ/VEH group (p < 0.01; Fig. 4A). However, although it approached significance, the one-way ANOVA did not show statistically significant effects on the total entries into all compartments of the social preference box in male mice (F(3,26 = 2.91, p = 0.053). Data are shown in Fig. 4C. Finally, there was an effect of treatment on the social preference index (F(4,64 = 0.69, p < 0.01). Planned comparisons revealed that male mice that received the combination TBZ/FLX increased the preference for the conspecific in comparison to the TBZ/VEH group (p < 0.01).

In females, the ANOVA showed significant effects of treatment on time interacting with the object (F(3,24) = 10.38, p < 0.01), and also on time interacting with the conspecific (F(3,24) = 3.41, p < 0.05). Planned comparisons revealed that female mice that received TBZ/FLX further reduced exploration compared to the TBZ/VEH group (p < 0.05 for the conspecific, and p < 0.01 for the object) (Fig. 4B). In addition, ANOVA also revealed a significant effect of treatment on total compartment entries (F(3,24) = 4.39, p < 0.05). Planned comparisons showed TBZ/FLX significantly decreased entries in comparison to the TBZ/VEH group (p < 0.01). Data are shown in Fig. 4D. Finally, there was no effect of treatment among females on the social preference index (F(3,24) = 2.07, p = 0.13).

### Experiment 4. Effect of bupropion or fluoxetine in TBZ-treated male and female mice on preference for sucrose concentration and on fluid consumption.

Groups of male mice (n = 47) and female mice (n = 46) received the same treatments as in previous studies and, during 60 min, were exposed concurrently to two bottles; one containing a solution of water with 5% sucrose and another bottle a 10% sucrose solution.

**Fig. 5:**
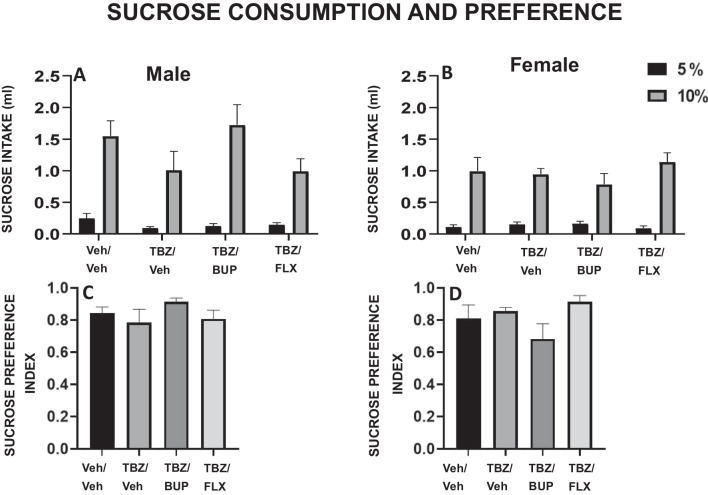
Effect of treatment on intake and preference for two different sucrose solutions (5 or 10%) during 60 min. Bars represent the mean ± S.E.M of milliliters consumed

The ANOVAs showed no significant effect of treatment on the total amount of 5% sucrose liquid consumed in male mice (F(3,44) = 1.81, p = 0.19) or female mice (F(3,43) = 0.95, p = 0.22). ANOVAs were also not significant for the 10% liquid sucrose consumed; in male mice (F(3,44) = 1.90, p = 0.14), and in female mice (F(3,43) = 0.75, p = 0.52) (Fig. 5 A-B). Finally, there were no significant effects of treatment on the sucrose preference index in either male (F(3,44) = 1.10, p = 0.35) or female mice (F(3,43) = 1.96, p = 0.13). Data are shown in Fig. 5 C-D.

## Discussion

The current studies examined how two different antidepressants with very different mechanisms of action (bupropion and fluoxetine) interact with the effects of TBZ, a DA depleting agent that in humans produces fatigue and depression (Frank 2009; Guay 2010; Chen et al. 2012), and that has been extensively studied and characterized in male rodent models of effort based decision making (Nunes et al. 2013b; Randall et al. 2014; Yohn et al. 2015, 2016; Pardo et al. 2015; Correa et al. 2018; Yang et al. 2020) and behavioral activation (López-Cruz et al. 2018; Carratalá-Ros et al. 2020, 2021a, b). Moreover, because TBZ has not been studied in females, the main interest of the present study was to assess, under the same experimental conditions in male and female mice, if TBZ could induce similar effects in females as it has previously done in males, and to evaluate if these two antidepressants can be effective at reversing those effects both in males and in females. Because we wanted the most statistically powerful way of assessing drug effects within each sex, we decided to analyze each sex separately, essentially conducting parallel but separate experiments in males and females. Disaggregating the data based on sex enhances the likelihood of detecting meaningful effects improving accuracy and avoiding misinterpretation of data (see reviews Tannenbaum and Day 2017; Tannenbaum et al. 2019). In addition, in order to reduce the number of animals (the 3R’s principles), we used the most effective doses and combination of drugs that were previously studied in males in a broader range of doses (Carratalá-Ros et al. 2021a, b a, b).

Thus, we assessed the effects of these two antidepressants after DA depletion in male and female CD1 mice on paradigms that evaluated depressive-like behaviors such as the FST, anxiety-like behaviors (DL box paradigm and EPM paradigm), avoidance of novel social stimuli (evaluated in a social interaction task) and, finally, preference and consumption of liquid sucrose that is often related to anhedonia-like behaviors. Our main hypothesis was that TBZ would have a major impact in mice of both sexes on those parameters that involve motivationally-induced behavioral activation to escape or avoid aversive and anxiogenic stimuli, but would have no effect on consummatory behaviors that do not involve behavioral activation.

In our experiments, male and female mice received TBZ, which acts by inhibiting VMAT-2), leading to a blockade of vesicular storage, and to a depletion of neostriatal and accumbens DA in rats and mice (Pettibone et al. 1984; Nunes et al. 2013a; López-Cruz et al. 2018). We used 8.0 mg/kg of TBZ since this dose has been shown to be effective for depleting ventral striatal DA (López-Cruz et al. 2018; Yang et al. 2020), as well as impairing in male mice behavioral activation on the FST (Carratalá-Ros et al. 2020, 2021a,b) impairing selection of effortful choices and behavioral activation in male mice tested on operant requiring exertion of physical effort, on the T-maze with RW, and also on the FST (Carratalá-Ros et al. 2020, 2021a,b; Yang et al. 2020). Thus, administration of 8.0 mg/kg TBZ induced behavioral activation impairments in both male and female mice in the FST, decreasing swimming behavior and increasing immobility. However, it only affected climbing (i.e., the most active behavior) in male mice, possibly because the baseline for climbing is very low in females (Fig. 1B.).

TBZ did not affect any of the anxiety parameters in either the DL box (time spent in the bright chamber), or in the EPM (time in the open arms) paradigms, in any of the two sexes. It seems worth mentioning that in the EPM female mice displayed lower levels of basal anxiety-related behavior and showed more active exploration than male mice. Consistent with previous findings (Correa et al. 2018; Carratalá-Ros et al. 2020), TBZ had a significant effect on voluntary exploration in the DL box (crosses between compartments) on male mice, but it did not have an effect on this parameter in female mice. Higher levels of locomotion in females and more anxious behavior in males has been reported in previous studies using the EPM in rats (Fernandes et al. 1999; Scholl et al. 2019; Knight et al. 2021).

The effect of TBZ was also evaluated for actions on social motivation using a social preference task. This kind of task allows the study of spontaneous motivation for social contact (in this case in non-isolated animals) manifested by social preference or avoidance, although it also has been used to evaluate anxiety in rodents (File and Hyde 1978; Guy and Gardner 1985; López-Cruz et al. 2016). In our experiments, male and female mice had to choose between interacting with a same sex-conspecific vs. spending time investigating an object. The present studies in mice, and previous studies in male and female rats, show that there are no differences between sexes in social exploration, although age affects females more than males (Perkins et al. 2016). We found that TBZ/VEH administration did not change social preference in male or female mice. In addition, the preference for different concentrations of liquid sucrose was also evaluated in male and female mice. Both sexes clearly preferred the higher concentrated solution (10% versus 5%), and TBZ/VEH did not change this preference, and, more importantly, did not reduce the total amount of fluid consumed. Previous studies also have shown no baseline difference between female and male rodents on liquid sucrose intake and preference (Dalla et al. 2008; Henderson et al. 2017). Sweet taste can act as a powerful natural reward for rodents (Levine et al. 2003; Yamamoto 2008), and preferences for higher concentrations (10%) of sucrose seem to invigorate behavior to work harder for those solutions, when a lower (0.3%) but freely available sucrose concentration is concurrently available (Pardo et al. 2015; San Miguel et al. 2018). A previous study in our laboratory using male rats, showed that TBZ only did induce a low effort bias in tests of effort-based choice when rats had to work on a lever pressing schedule to obtain the highly concentrated sucrose but this effect was not seen when rats had concurrent but free access to both concentrations of sucrose (Pardo et al. 2015), indicating TBZ did not produce any change in concentration preference or the total volume consumed, consistently with our present results in mice. More recent research has shown that TBZ administration did not alter intake of highly palatable chocolate in rats tested on a binge-like eating task (Salamone et al. 2022), and did not produce any change in sucrose concentration preference or the total volume consumed (Pardo et al. 2015), consistently with our present results in mice.

Thus, taken together from present and previous work, it seems that administration of TBZ, which depletes accumbens DA in both rats and in mice (Nunes et al. 2014; López-Cruz et al. 2018; Yang et al. 2020), does not affect aspects of sucrose motivation such as intake, preference or hedonic reactivity when the effort requirement for obtaining sucrose was minimal (Pardo et al. 2015). However, selection of vigorous instrumental behaviors reinforced by sucrose is sensitive to the effects of TBZ, which indicates that DA regulates behavioral activation, leaving intact the directional aspect of motivation (approaching to a palatable reward when effort is not required; Pardo et al. 2015). On the whole, these results confirm the idea that drugs that modify DA transmission do not affect emotional parameters related to food preferences, anxiety or sociability. Both sexes show similar baselines, at least when they are young and middle-aged adults.

Because the effects produced by TBZ in male and female mice on different behavioral paradigms are often used to measure depressive-like behaviors (Randall et al. 2015; Yohn et al. 2015, 2016), the next step was to observe if two antidepressants with different mechanisms of action (bupropion and fluoxetine) were able to reverse the behavioral impairments induced by TBZ, and if there were different patterns of effects in males and females in terms of the response to these two types of drugs. BUP is a catecholamine reuptake inhibitor that acts on DAT and elevates extracellular DA in Nacb (Randall et al. 2015). In humans, BUP has demonstrated to treat effectively some symptoms of depression (Feighner et al. 1986; Kiev et al. 1994; Weihs et al. 2000; Papakostas et al. 2006; Pae et al. 2007; Cooper et al. 2014), and also to increase active behaviors in animal models that evaluate depressive-like behaviors (Yamada et al. 2004; Kitamura et al. 2010; Yuen et al. 2017; Carratalá-Ros et al. 2021b). However, little is known about the effect of BUP on female rodents. In the present studies, the administration of 10.0 mg/kg of BUP, a dose that is effective at increasing active behaviors in male mice assessed in the FST (Carratalá-Ros et al. 2021b), alleviated the behavioral effects of TBZ in both sexes, increasing time spent swimming and decreasing immobility. However, BUP only was able to increase climbing behavior after TBZ administration in male mice (Fig. 1). As expected, the combination of TBZ with BUP did not have any effect on anxiety-like behaviors measured as time spent in the bright chamber of the DL box, and time spent in the open arms of the EPM (Fig. 2A-B). These results are in accordance with previous studies done in our laboratory in which BUP alone or in combination with TBZ did not modulate any parameter related to anxiety-like behaviors in male mice (Carratalá-Ros et al. 2021b).

FLX primarily functions as an inhibitor of the serotonin transporter (SERT), preventing uptake of serotonin (Nutt 1999; Alex and Pehek 2007). In humans, this antidepressant is useful for treating emotional symptoms present in depression (Papakostas et al. 2008; Rosenblau et al. 2012; Rizvi et al. 2013; Hieronymus et al. 2016). However, FLX seems less effective for treating motivational dysfunctions, and in fact, it can exacerbate or induce these symptoms, such as fatigue, in some patients (Nutt et al. 2007; Targum and Fava 2011; Padala et al. 2012; Stenman and Lilja 2013; Fava et al. 2014; Rothschild et al. 2014). In previous studies using the FST, an aversive test, the administration of FLX alone increased active behaviors (swimming and climbing) and decreased immobility in male rodents (Petit-Demouliere et al. 2005; Jang et al. 2009; Castagné et al. 2010; Carratalá-Ros et al. 2021a), and also in a few studies using female rodents (Jones and Lucki 2005; Fernández-Guasti 2017). However, in a positive context, FLX administered alone has been demonstrated to impair effortful behaviors, such as lever pressing to obtain access to food, and to reduce voluntary RW activity in male mice (Carratalá-Ros et al. 2021a), and in male and female rats (Presby et al. 2021). Thus, FLX alone improved scaping behaviors, but it did not induce an active approach to positive stimuli. However, when behavioral activity was impaired by TBZ, FLX was not able to reverse, and even exacerbated, the impairments produced by TBZ in both type of contexts; the FST and the T-maze RW choice task in male mice (Carratalá-Ros et al. 2021a). Interestingly, in the present results the administration of FLX plus TBZ had a different pattern of effects in male vs. female mice. As expected, FLX in males did not improve TBZ-induced impairments in the FST, but in females this SERT blocker reversed the effect produced by TBZ in swimming and immobility, in a similar way to BUP. However, it is unlikely that this improvement was due to anxiolytic effects since, in our studies, females showed some anxiogenic effects after FLX plus TBZ administration, reducing time in the open arms of the EPM, and reducing also time interacting with both stimuli (object and conspecific) in the social test. Thus, FLX in TBZ treated females potentiated active escape, but also passive avoidance of potentially threatening conditions.

This combination of drugs (TBZ + FLX) reduced locomotor exploration in the DL, the EPM and also the social interaction chamber in females (see Figs. 2D, 3D, and 4F). Previous studies have reported that FLX in male rats and mice that had received TBZ showed further decreases in locomotor activity compared with administration of TBZ alone (Podurgiel et al. 2015; Carratalá-Ros et al. 2021b). However, as a whole, the main effects of these drugs on emotional and motivational parameters do not seem centrally linked to voluntary locomotion results in those same tests since in some cases locomotion increases (Fig. 3C) and in others it decreases. These results are in agreement with the observation that SERT antidepressants are relatively ineffective for treating activational dysfunctions, and, they may exacerbate or induce these symptoms in some patients (Nutt et al. 2007; Targum and Fava 2011; Padala et al. 2012; Stenman and Lilja 2013; Fava et al. 2014; Rothschild et al. 2014).

In summary, the present experiments provide information about the behavioral performance of female mice under basal conditions, and also after TBZ administration, in different animal paradigms that evaluate emotional and motivational components of depression-like behavior. Our results indicate that TBZ affects behavioral activation, and based on other lines of evidence it is likely that there is a large dopaminergic component to this aspect of TBZ actions (Yohn et al. 2016; Salamone et al. 2022). This suggests that pro-dopaminergic drugs may be able to improve depressive symptoms related to reduced fatigue and lack of energy in humans (Salamone et al. 2022). Moreover, the differential patterns of pharmacological effects shown in male and female mice support the differential efficacy of distinct monoamine uptake inhibitors in the treatment of specific motivational symptoms in both sexes. Mouse models of depressive symptoms show sexually divergent networks in the brain with distinct patterns of stress-induced gene regulation in males and females. These findings have now been reproduced in human postmortem tissue and may provide insights into why males and females with major depressive disorder respond differently to treatment with antidepressants (Tannenbaum and Day 2017; Tannenbaum et al. 2019). These ideas are consistent with the research domain criterion (RDoC) approach that highlights the importance of describing the neural circuits that mediate specific symptoms in psychopathology, and not simply the traditional diagnostic categories (Cuthbert and Insel 2013; Salamone and Correa 2022). Furthermore, it highlights the fact that relatively limited basic research has been devoted to developing animal models and consequently describing drug treatments which are sensitive to sex differences. These results suggest caution should be exercised in interpreting the results from female rodents in tests validated on males.

## Data Availability

Any data will be made available upon reasonable request to the corresponding author.
